# The Amenities of Statistics

**Published:** 1861-07

**Authors:** 


### Art. IV.—THE AMENITIES OF STATISTICS.

It will doubtless be no easy task for many to conceive the possibility of statistics being in any way associated with aught that is agreeable. The very mention of the term is apt to call up dread visions of figures and tables, before which the most hilarious spirit might quail.* Nevertheless, it is a sober truth that even

* "Numbers lie at the foundation of statistics as well as of the other exact sciences. But it would be as great a mistake to assume that figures and tables statistics are highly capable of being made an efficient means unto an enlivening end, and that statisticians of the purest blood, notwithstanding the peculiar disheartening circumstances under which their lives, according to popular views, might be supposed to be passed, may retain, like the celebrated Mr. Mark Tapley, an exceeding jollity of disposition under those circumstances.

It has become the pleasant custom of the leading statisticians of the chief civilized countries in the world, to meet together from time to time under the auspices of their respective governments. After the happy example of the British Association for the Advancement of Science, these meetings are equally conducive to pleasure and relaxation and the promotion of Statistical Science. Under their influence the science becomes hospitable, and hospitality becomes scientific, and each gains from being brought into intimate association with the other.

The idea of these gatherings of statisticians originated in London at the time of the Great Exhibition of 1851, and the first, under the title of International Statistical Congress, was held in Brussels in 1853. The second Congress met in Paris in 1855; the third in Vienna in 1857; and the fourth in London in July last. The Report of the proceedings of the last Congress (printed by Government, and edited by Dr. Farr) has been recently published, and we propose briefly to touch upon those matters contained in it which we conceive would be of most interest to our readers.*

The Prince Consort presided, and opened the session with one of those happy and well-conceived addresses for which he has become so famed. After welcoming the foreign delegates to this country, he pointed out that Statistical Science was earliest developed here. We are indebted for this honour, Dr. Farr tells us, to Sir William Petty, a physician, born at Romsey, in Hampshire, on 26th May, 1623. He "studied medicine in Paris, in the Netherlands, and at Oxford; and by practising physic, measuring forfeited lands, and speculating in them, he, more fortunate than his successors, amassed a fortune of a quarter of a million. His Essays in *Political Arithmetic* laid the foundation of statistics. He died in 1687."

His Royal Highness then, in the following language, skilfully

---

express the whole doctrine of statistics, as to suppose that nautical almanacks embody the whole science of astronomy. The sublimest considerations arise out of the numerical laws, expressing either the relations of the parts of the universe, or the relations of the successive generations of men."—Dr. Farr.

* Official delegates were present at the fourth Congress from Austria, Bavaria, Belgium, Brazil, Denmark, France, Hamburg, Lubeck, and Bremen, Hanover, Holland, Mecklenburg-Schwerin, Norway, Prussia, Russia, Sardinia, Saxe-Coburg and Saxe-Meiningen, Spain, Sweden, Switzerland, Turkey, the United States, several of the British colonies, and other countries connected with Great Britain.

touched upon the prejudices which so largely exist on the subject of statistics:—

"Gentlemen," he said, "old as your science is, and undeniable as are the benefits which it has rendered to mankind, it is yet little understood by the multitude; it is new in its acknowledged position among other sciences, and still subject to many vulgar prejudices.

"It is little understood, for it is dry and unpalatable to the general public in its simple arithmetical expressions; representing living facts (which, as such, are capable of arousing the liveliest sympathy) in dry figures and tables of comparison. Much labour is required to wade through endless columns of figures, much patience to master them, and some skill to draw definite and safe conclusions from the mass of material which they present to the student; while the value of the information offered depends exactly on its bulk, increasing in proportion with its quantity and comprehensiveness.

"It has been little understood, also, from the peculiar and often unjustifiable use which has been made of it; for the very fact of its difficulty, and the patience required in reading up and verifying the statistical figures which may be referred to by an author in support of his theories and opinions protect him to a certain extent from scrutiny, and tempt him to draw largely upon so convenient and available a capital. The public, generally, therefore, connect in their minds statistics, if not with unwelcome taxation (for which they naturally form an important basis), certainly with political controversies, in which they are in the habit of seeing public men making use of the most opposite statistical results with equal assurance in support of the most opposite arguments. A great and distinguished French minister and statesman is even quoted as having boasted of the invention of what he is said to have called '*l'art de grouper les chiffres;*' but if the same ingenuity and enthusiasm which may have suggested to him this art should have tempted him or others, as historians, to group facts also, it would be no more reasonable to make the historical facts answerable for the use made of them, than it would be to make statistical science responsible for many an ingenious financial statement.

"Yet this science has suffered materially in public estimation by such use, although the very fact that statesmen, financiers, physicians, and naturalists seek to support their statements and doctrines by statistics, shows conclusively that they all acknowledge them as the foundation of truth, and this ought therefore to raise instead of depressing the science in the general esteem of the public."

On the objections which have arisen out of the seeming incompleteness of statistical science in its aims, and its being, as it were, subsidiary to other sciences, rather than a science itself, His Royal Highness observed:—

"But this is in appearance only; for if pure statistics as a science abstains from participating in the last and highest aim of all science—viz., the discovery and expounding the general laws which govern the universe, and leaves this duty to its more favoured sisters—the natural and the political sciences—this is done with conscious self-abnegation, for the purpose of protecting the purity and simplicity of its sacred task—the accumulation and verification of facts, unbiassed by any consideration of the ulterior use which may or can be made of them."

The Prince Consort made, moreover, a vigorous protest against the notion that statistics necessarily lead "to Pantheism and the destruction of true religion;" and he further remarked:—

"But we are met, also, by the most opposite objections, and statistics are declared useless because they cannot be relied on for the determination of any given cause, and do only establish probabilities where man requires and asks for certainty. This objection is well founded, but it does not affect the science itself, but solely the use which man has in vain tried to make of it, and for which it is not intended. It is the essence of statistical science that it only makes apparent general laws, but that these laws are inapplicable to any individual case; therefore, what is proved to be law in general is uncertain in particular. Herein lies the real refutation, also, of the first objection [that of pantheistic tendency], and thus are the power, wisdom, and goodness of the Creator manifested; showing how the Almighty has established the physical and moral world on unchangeable laws conformable to His eternal nature, while He has allowed to the individual the freest and fullest use of his faculties; vindicating, at the same time, the majesty of His laws by their remaining unaffected by individual self-determination."

The opinion that statistical science tends to sap the foundation of religious belief, is of such serions import, and has of late been apparently so conspicuously confirmed by the writings of one of the most popular historians of the day, that we shall be pardoned quoting at length (from the the Report upon the Programme of the Congress) the following admirable exposition by Dr. Farr of the fallacy upon which the opinion rests.

"It is the characteristic of the primary statistical phenomena that they are all of vital interest. And it has been found that they are governed by laws; so that a deduction from a given series of observations admits of general application. The past enables us to forecast the future; and the statistical discoveries of one nation are the lights of all nations. If conflagrations break out in cities and destroy a certain number of houses in a series of years, the same number of houses, in proportion, may be expected to be destroyed by fire in the following years, and in other cities. The proportional value of various kinds of property which is destroyed by fire annually fluctuates within assignable limits. The losses at sea in past years enable underwriters to estimate the losses of the coming year, and to provide the necessary insurance. If out of 100,000 children born, 50,000 have been found to live forty-five years, it is probable that out of 100,000 other children the same numbers will survive the same number of years; indeed, it is probable that the same numbers, or numbers only varying within given limits in the two series, will die year by year until the two generations are extinct. Thus, despite the accidents of conflagrations, the unstableness of the winds, the uncertainties of life, and the variations of men's minds and circumstances, on which fires, wrecks, and deaths depend, they are subject to laws as invariable as gravitation, and fluctuate within certain limits, which the calculus of probabilities can determine beforehand. Upon this basis the great system of insurance rests securely. And all statistical facts have the same character; the phenomena observe a definite order, and the same acts of bodies of men are found by experience to recur with the same intensity under the same circumstances. This holds of crimes, and other acts of the will; so that volition itself is subject to law.

"Shall a system of fatalism be built upon this foundation? Shall it be inferred, because statistical phenomena are governed by fixed laws, that man is a mere spectator of historical phenomena over which he has no real control? Can he study the past and speculate upon the future, but effect no change in the destinies of his race? Are statists to found a philosophy which abandons nations to Fate?

"No; for statistics has revealed also a law of variation. The number of houses that are burnt down in a large city usually varies little from year to year while their structure is the same; but if you substitute brick or stone for wood, and stipulate for party-walls, conflagrations still occur regularly, but at rarer intervals. Cities are not burnt to the ground; and the insurance premiums are reduced. Under one system of working, mining operations kill *eight* in a thousand men annually; under another, *four;* and in both systems the proportion over a series of years ranges within limits determinable mathematically. Introduce a system of ventilation into unventilated mines, and you substitute one law of accidents for another. So with regard to ships: some seas are calm, others are treacherous and stormy; different voyages have their several dangers, which can be expressed in numbers; so much per cent. of merchandise is lost on an average. But the dangers of the seas are not uncontrollable. With ill-constructed ships, and bad seamen, you lose a certain amount of human life and merchandise; in good ships, well manned, handled by skilful pilots, on well-lighted coasts armed with life-boats, you still lose life and merchandise by shipwreck, but in greatly diminished proportions. You substitute one law of loss for another. Therefore these events are under control. So the first ocean navigators perished in great numbers. A half, a fourth, a tenth of the crews, died once by scurvy and fever on long voyages; now the rate of mortality is less than *two* per *cent.* in Her Majesty's navy. Cities were ravaged by plagues in the middle ages; and, until quite recently, their inhabitants died at the rate of four and five in one hundred annually. In London, little more than *two* in one hundred of the population died in the year 1859. Human life has in it, indeed, a fatal element. No man lives beyond a certain age. It is exceedingly improbable that any given person will live over the age of one hundred years. A century is the secular cycle of human life. But it is found that numbers of a generation of men die at all the intermediate points of time in their allotted century of lifetime; their course is never completed. The chance of living any part of that time is determinable, and can be expressed by a fraction, like any other probability. Under given conditions the average duration of lifetime is forty-nine years; under other conditions it is twenty-five years. The conditions remaining the same, the lives of successive generations of men, as they succeed each other, describe the same average number of years; just as under the same winds the waves would break in equal numbers on the shores of the ocean. That is the law of life. The lifetimes also vary. The chances of life change. So also do the conditions; and the determination of those conditions on which the variations depend is the great problem which statistical inquiry seeks to solve, and has already succeeded in solving in a certain number of cases. Then it is true that crimes are committed with appalling regularity; they recur in the criminal returns year by year, and at every year of age; so definite numbers of successive generations of men commit similar crimes in similar ways; and it may be inferred from analogy that the majority of men discharge acts of duty, piety, and beneficence with the same constancy. Some races, however, commit crimes of violence in greater proportion than other races. Some classes of the same nation commit theft more frequently than others. Everything else being equal, criminal acts are proportional to the temptations; diminish the opportunities for crime and you diminish crime. The Guarantee Societies vary their premiums definitely according to circumstances. Under the prevalent system of conducting business with imperfect accounts, commercial companies lose a certain percentage by fraud; under a better system the frauds will be diminished. The laws of fraud vary. The products of industry also increase as the rewards of industry increase. The theory of rewards and punishments implies that the mind is under control. As men, then, have the power to change the conditions of life, and even to modify their race, they have the power to change the current of human actions, within definite limits, which statistics can determine.

"Statistics enables us to exhibit in definite numbers the great facts of national life; it also determines the laws of life and action. These laws are constant, under the same circumstances; and their discovery supplies the means—as insurance—of mitigating inevitable evils. The utility of statistics is still further displayed in its determination of the law of variations, under varying circumstances; as it points out directly the modifiable causes of life and death, of good and evil, of happiness and misery, of national decay and of national prosperity."

On the aim of the Congress the Prince Consort thus spoke, and his observations form a fitting epigraph to the report:—

"It is the social condition of mankind, as exhibited by [statistical] facts, which forms the chief object of the study and investigation undertaken by this Congress; and it hopes that the result of its labours will afford to the statesman and legislator a sure guide in his endeavours to promote social development and happiness. The importance of these international Congresses in this respect cannot be averrated. They not only awaken public attention to the value of these pursuits, bring together men of all countries who devote their lives to them, and who are thus enabled to exchange their thoughts and varied experiences ; but they pave the way to an agreement among different governments and nations to follow up these common inquiries in a common spirit, by a common method, and for a common end."

Proceeding now to the general business of the Congress, among those subjects which come within the sphere of our Journal, we would first direct attention to the report on the statistics of literature. This claims the attention and must prove of great interest to every practical psychologist. The report was drawn up by Mr. Winter Jones, of the British Museum, and it is couched in a series of propositions which were unanimously adopted by the Congress. The first two propositions run thus, and they will suffice to show the chief objects aimed at, and their bearing upon psychology :—

"(1) The literary statistics of a country ought to embrace all that is the result of the exercise of the human intellect, so far as the same is manifested through the Press. The most ephemeral street-ballad must find a place in its details, no less than the work of the highest scientific character. The Press is called into operation so generally, its use is so necessary for the diffusion of information, so indispensable for the successful accomplishment of many of the most important transactions of life, that its statistics embraces perhaps a wider field than that of any other branch. It affords an index to the material, intellectual, and moral condition of a nation, and if carried sufficiently far, will show the special character of the industry of every country.

"(2) Care ought, however, to be taken to give to its details as little of a critical character as possible. It is very important to know that a particular country has published, in the course of a year, fifty works on palæontology ; but if the contents or character of each were to be given, the result would be an amount of materials, collected at an expense of time and labour, which would soon prove fatal to the undertaking."

The subsequent propositions refer to the definition of terms, and the method of compiling the statistics ; and they enumerate for inquiry, in addition to new works and volumes published annually, the numbers (1) of new editions; (2) of books published in different languages; (3) of authors ; (4) of anonymous publications ; (5) of reprints of foreign works ; (6) of printers, showing the number of men employed, or the number of printing presses; (7) of publishers, subdivided according to the particular class of books published by them, as far as possible, showing the number of works imported by them into each country, and the number exported from each country, in each class; (8) of second-hand booksellers ; (9) of book auctioneers ; (10) of circulating libraries ; (11) of libraries attached to learned institutions, educational and scientific; (12) of public libraries; (13) of volumes in the above libraries, classified according to a method suggested; also the regulations for admission to and use of the same libraries, with the number of readers and of volumes used by them; (14) of works and volumes published by the Government, or by the aid of Government support, whether such support be in the form of a sum of money or by taking a certain number of copies; and, (15) the number of works, the publication or sale of which has been prohibited by the Government or by the Church.

In presenting the report to the Congress, Mr. Monckton Milnes pithily observed that "the Statistics of Literature are in truth the complement and the crown of the Educational Statistics of a country. We can show by Educational Statistics what we teach, and we may show by our books what we have learnt."

In countries where a compulsory deposit of books exists, such as the *dépot légal* in France, and the deposit of books at the British Museum, the compilation of literary statistics, so far as books are concerned, is quite possible, and it is proposed that the Governments of these countries should be requested to cause lists of such books to be printed. The possibility of carrying out several of the remaining heads of inquiry may also be admitted, although of others it is for the present questionable.

The propositions adopted by the Congress in reference to Army and Navy Vital Statistics, and Military Sanitary Statistics are of great interest. The vast practical value of such statistics has been conclusively shown in our own army, and their scientific importance could hardly be exaggerated. The circumstances under which both soldiers and sailors are placed are peculiarly available for solving some of the most important problems in the development, spread, and geographical distribution of diseases. From the facilities possessed in armies and navies for determining the various influences to which the men are exposed, and the probable effect of those influences, the great and complex questions of health and disease are, among soldiers and sailors, reduced perhaps to the greatest simplicity of which such questions, as a rule, are capable. But these advantages can prove of no avail unless connected with a well-conceived and permanent method of statistical observation. To impress this truth upon different Governments will be one of the most valuable of the services rendered by the International Statistical Congress. Our own Government and that of the United States have alone become as yet alive to the important fact. Since 1835 the military authorities in this country have been becoming more and more conscious of the great advantages which would arise from a system of army vital statistics, and whatever doubts might have remained, these were pretty effectually swept away by the events which transpired during the Crimean war. It is the intention of the English Government in future to publish an Annual Statistical Report of the Health of the Army. The first report is now in course of preparation.

In illustration of the practical utility of statistical health reports, we may mention two facts stated by Dr. Balfour during the discussion in the Congress upon the Vital Statistics of the Army. Five statistical reports upon the health of the army, in different quarters of the globe, were published, within the fifteen years 1838-1853, and in consequence of the improvements adopted upon the suggestions contained in and arising out of these reports, the mortality in Jamaica, "which in the previous twenty years had averaged 140 per 1000 of the strength, has been reduced so much, that during the last four or five years, it has only averaged 30 per 1000;" and "the mortality in Ceylon, which had amounted to 75 per 1000, has averaged during the second period 38 per 1000."

The propositions agreed to by the Congress were drawn up by Dr. Balfour, Dr. Bryson, and Dr. J. Sutherland, and they form an admirable basis for a uniform system of Army and Navy Vital, and Military Sanitary, Statistics.

The section on Sanitary Statistics was occupied chiefly with discussing (1) a proposal for a uniform plan of hospital statistics by Miss Nightingale; (2) certain heads of inquiry for sanitary statistics suggested by Dr. Sutherland; and (3), a scheme of general sanitary statistics by Dr. Farr.

The advantages sought to be obtained by Miss Nightingale's proposal are best set forth in her own words:—

"Up to the present time the statistics of hospitals have been kept on no uniform plan. Every hospital has followed its own nomenclature and classification of diseases, and there has been no reduction, on any uniform model, of the vast amount of observations which have been made in these establishments. So far as relates either to medical or sanitary sciences, these observations in their present state bear exactly the same relation as an indefinite number of astronomical observations made without concert, and reduced to no common standard, would bear to the progress of astronomy. The material exists, but it is inaccessible.

"With the view of rendering the present stores of observation useful, and of collecting all future observations on one uniform plan, tables have been prepared for recording, on one common form, all the facts of hospital experience.

"They have been already tried in several hospitals, and the results have been sufficient to show how large a field for statistical analysis and inquiry would be opened by their general adoption.

"They would enable us to ascertain the relative mortality in different hospitals, as well as of different diseases and injuries at the same and at different ages, the relative frequency of different diseases and injuries among the classes which enter hospitals in different countries and in different districts of the same country. They would enable us to ascertain how much of each year of life is wasted by illness—what diseases and ages press most heavily on the resources of particular hospitals. For example, it was found that a very large proportion of the limited finances of one hospital was swallowed up by one preventible disease—rheumatism—to the exclusion of many important cases or other diseases from the benefits of the hospital treatment.

"It has been shown that most of the cases admitted to the hospitals, where the forms have been tried, belong to the productive ages of life, and not to the ages at the two extremes of existence.

"The relation of the duration of cases to the general utility of a hospital has never yet been shown, although it must be obvious that if, by any sanitary means or improved treatment, the duration of cases could be reduced to one-half, the utility of the hospital would be doubled, so far as its funds are concerned.

"The proposed forms would enable the mortality in hospitals, and also the mortality from particular diseases, injuries, and operations, to be ascertained with accuracy; and these facts, together with the duration of cases, would enable the value of particular methods of treatment and of special operations to be brought to statistical proof. The sanitary state of the hospital itself could likewise be ascertained. The statistics of rare diseases and operations are still very imperfect; but by abstracting the results of such diseases and operations from the tables after a long term of years, trustworthy data could be obtained to guide future experience."

These lucid and valuable observations need no comment on our part. Miss Nightingale's proposal gave rise to an animated discussion, and many suggestions were made, several of which were adopted by the Section and assented to by Miss Nightingale. The practical result of these additions would be "to leave," as the distinguished Reporter of the Section, Dr. McWilliam, C.B., states, "the forms proposed by Miss Nightingale, and adopted by the Section, as they are, except that an explanatory direction will have to be given on the margin, to point out how certain of the proposals may be incorporated in recording cases. The others will have to be made subjects of separate record in hospital books, from which they will have to be separately abstracted for comparison with the data tabulated on the forms."

The thanks of the Congress were given to Miss Nightingale for the great trouble she had taken in this important matter; and to mark its sense of the value of her propositions, the Congress recommended that they should be adopted by the Section and made known to the different Governments.

A form for separating diseases from persons on the hospital record, prepared at the request of the Section, by Dr. Berg, the Director of the Statistical Department of Sweden, was also adopted.

Dr. Sutherland's *Heads of Inquiry for Sanitary Statistics,* with the modifications adopted by the Congress, are calculated to prove a valuable aid to the sanitary observer, and will go far to secure a scientific registration of the facts connected with health and disease. His preliminary and concluding observations may be quoted with advantage:—

"A Statistical Inquiry into the sanitary state of any population includes much more than the annual proportion of deaths, and the nature of those diseases which have occasioned the mortality.

"In Sanitary Statistics facts and conditions must go together. We must know the nature of the population, its proportions of ages, sexes, and occupation, before we can compare its mortality statistics for any practical purpose with those of other populations. We must also ascertain by inquiry on the spot *where* the mortality has occurred. This is a very important point. In some parts of a town, the proportion of young children may be much larger than in other parts; and this fact alone would in the former cases determine a higher rate of mortality and a lower mean duration of life than in the latter. Some districts of towns are in a much worse sanitary state than other districts, and would furnish on that account a higher rate of mortality. Before the sanitary state of Liverpool was improved, its mortality, taken over the whole population of the parish, was 1 in 28·75 per annum; but when the fact was examined in detail, it was found that the annual mortality in the healthiest ward was 1 in 41·62, while in the most unhealthy ward it was 1 in 23·50 of the populations.

"Again, the average age of death in the same town was found to be 17; but when this fact was examined in detail, it was found that in the healthiest ward the average age of death was 22·57 years, while in the most unhealthy ward it was 14·93 years.

"But to arrive at the truth, the inquiry must be carried much further. Individual streets must be taken, individual houses must be taken, and even individual parts of houses. By pursuing this method of inquiry, not only are towns compared with towns, but streets with streets, and houses with houses, in the same town. It has been made matter of statistical proof that particular streets, or courts, certain houses, and even certain flats of houses, are less healthy and more predisposed to particular diseases than are other streets, houses, or flats.

"The basis of all true Sanitary Statistics is hence street to street and house to house inquiry. It is in this way that such trustworthy facts can be elicited, as, when compared with other equally well-ascertained facts, can lead to practical sanitary improvement."

Dr. Sutherland next proceeds to indicate those points which influence the sanitary condition of a population and which admit of statistical classification, and he concludes his suggestions by observing that—

"The time appears to have arrived when accurate sanitary statistics should not only be kept for all branches of the public service, but also by all corporations, municipalities, boards of commissioners, and parish vestries, for the population within their respective jurisdictions. The whole could be done by a few active individuals, who would take sufficient interest in the matter; and it is to be hoped that anybody, corporate or municipal, will, at no distant period, publish the entire statistics of its population, and especially its sanitary statistics, once every year.

"At present this class of facts, which it is of the highest interest for every community to know, is all but unknown. The facts are there, ready to be classified; all that they want is careful observation and accurate recording. It is by doing so alone, that the natural history, so to speak, of any population can be ascertained.

"The machinery exists. It only requires to be directed. There are officers of health, inspectors, poor-law medical officers, registrars, and private individuals, who would, no doubt, willingly lend their aid in so great a work, if it were undertaken on some general plan."

Dr. Farr's comprehensive scheme for determining the sanitary condition of the population of all civilized states, "contains (to quote the language of the sectional report) very important propositions for arriving at the following objects:—

"First, an accurate statistical estimate of the comparative healthiness or unhealthiness of districts or circumscriptions, having defined and acknowledged boundaries in each nation, as shown by the rate of mortality per 1000, the mean duration of life, and the chief fatal diseases in each district. By the same comparisons the healthiness of the whole nation may be estimated and compared with that of other nations. An important element in the same inquiry is the number of persons suffering from illness sufficient to prevent them following their customary occupations, which Dr. Farr proposes to obtain through the medium of the census, hospitals, friendly societies, and other similar channels.

"Second, to carry the inquiry into the physical state of the population, as indicated by stature, weight, strength, intelligence, indications of health, &c., among groups of the population.

"Third, to include in such a survey the influence of locality, habitation, and occupation on health, with regard to the last of which elements, Dr. Farr has given certain forms of tables whereby the facts may be easily arranged.

"The remaining propositions refer to certain recommendations which were discussed fully by the Section, referring to the publication, at regular intervals, of returns of births, marriages, and deaths; accounts of prevailing epidemics, the appointment of officers of health, and the publication, at frequent intervals, of the reports of such officers.

"The object of the whole being to collect accurate and uniform facts bearing on the health and physical well-being of populations, the results of improvements, as well as the consequences of neglects, and to diffuse the information so obtained for the common benefit."

"The Section," it is added, "cordially adopted Dr. Farr's recommendations, with one or two slight modifications involving more frequent publication of certain reports than was recommended in Dr. Farr's proposals."

In addition to the subjects especially set apart for discussion in the Programme of Proceedings, several papers and propositions were submitted to the Sanitary Section. Mrs. Baines directed attention to the great evils arising from "wet nursing," and made several ingenious suggestions by which, through obstetrical practitioners, medical officers of health, and registrars, more trustworthy information might be obtained on the subject. Dr. E. Jarvis, the president of the American Statistical Association, submitted an important proposition, which was adopted by the Section, for a uniform system of Reports in Lunatic Asylums. A resolution was carried to communicate Dr. Jarvis's proposition to the Commissioners in Lunacy,* the Society of Medical Officers for the Care and Treatment of the Insane, and to the several authorities in such matters in all parts of Europe. Dr. Milroy read a highly interesting paper, containing several valuable suggestions, on the "International Registration of Epidemics." He observed that—

"Hitherto but little has been done in the way of observing and registering the geographical and chronological development and distribution of these diseases over extensive regions of the globe. The researches of inquirers having to the present time been necessarily confined to their own country, it is obvious that, unless like researches are being carried on simultaneously in other countries, contiguous and more remote, some most interesting problems of epidemiology, such as the migratory course of certain epidemics, their recurrence at irregular intervals, their subsidence at one time, and their reappearance at another, can never be hoped to be elucidated. It is worthy of note, that the last sixteen or eighteen years have afforded some most remarkable illustrations of the kind—viz., not only the far more extensive diffusion of the cholera since 1847 than previously, but also the total cessation since 1848 of the plague (with the exception of the recent local outbreak at one part of the Barbary coast) throughout the Ottoman dominions, likewise the springing up in 1849 for the first time of a great epidemic of yellow fever in the Brazils, thence spreading gradually over the entire Gulf of Mexico and West Indian Archipelago, till it reached some of the northerly provinces of the United States.

"Now, what has been done of recent years with so much advantage by the synchronous observation and record in different regions of the phenomena of meteorology, magnetism, and other branches of physical sciences, might probably be undertaken with similar benefit by the registration, on the same plan, of epidemic statistics."

Dr. Milroy, then, after expressing an opinion that there was

* This recommendation was added to the resolution at the suggestion of the President of the Section, the Right Hon. the Earl of Shaftesbury, the President of the Lunacy Board.

more connexion between the development of an epidemic disease in different countries and between the successive appearance of different diseases than has been generally imagined, suggested that a system of synchronous observation might probably be instituted by aid of the Congress. He indicated the diseases which in the first instance should form objects of attention, and those points of their history which specially deserved to be noted.

Mr. Ernest Hart introduced the important subject of the registration of sickness, and it was resolved "that the co-operation of all the different Governments be requested in collecting and publishing returns of sickness and meteorology in their several capitals." Dr. Greenhill directed attention to certain fallacies existing in the statistics of societies for improving the dwellings of the poor under their present form, and offered some suggestions by which those fallacies might be removed, and the establishment of such societies promoted. Professor Simonds, of the Royal Veterinary College, drew attention to the desirability of adopting means for ascertaining the extent and fatality of epizoötics and other diseases among animals which are ordinarily used for food. A resolution to that effect, as well as that the authorities should be recommended to obtain the information by appointing for that purpose veterinary and other officers of a similar kind, was agreed to.

We have touched upon many of the points in this remarkably valuable Report of the Congress, which we conceive might prove of interest to our readers, but we are far from having exhausted, even by enumeration, the subjects which come within the province of our Journal, and which are scattered in the body of the Report and in the Appendix. In the former, many interesting and important facts, immediately bearing upon the physical and moral condition of man, are to be found disseminated throughout the different discussions and reports; and in the latter are several most valuable and suggestive papers. We may notice, for example, Mr. Gompertz's paper, "On one Uniform Law of Mortality from Birth to Old Age, and on the Law of Sickness," Mr. T. R. Edmonds's observations on the Statistics of Health, and Dr. Jarvis's deductions on the comparative liability of males and females to various kinds of crime. These deductions are of singular interest, and they are derived from the records of somewhat over 65,000 committals to prison in which the sex and crime were given.

"I analysed these," Dr. Jarvis writes, "into two classes, first of the two sexes, and next of the crimes committed by each, and then determined the proportion of each crime to the whole number of crimes of each sex, and then compared these with each other, with the following result:—

"Among these criminals and convicts thus presented to me, most of violations of the law by males were crimes against persons and against property.

"Most of the females were committed for crimes of intemperance, lasciviousness, night-walking, &c.

"A majority of the males were committed for crimes that grew out of their intellect, and which required thought, plan, and reason for their execution.

"A majority of the females were committed for crimes that grew out of their physical organization—the indulgence of their appetites and bodily passions.

"A majority of the male crimes were those in which the violator of the law determined to benefit himself and to injure another.

"A majority of the female crimes were those in which they had no intention of injuring others, but in which they were inevitably sure to injure themselves.

"A majority of male crimes were crimes of selfishness.

"A majority of female crimes were crimes of self-sacrifice.

"From the same cause or origin of crime, the inducements to transgression for which most of these females were imprisoned, are more permanent and less under their own control, when once yielded to, than those which lead the males into error.

"Passion and appetite being inherent or excited in the animal constitution, or permitted to govern the individual to whom they belong, are not so easily nor so commonly repressed by the ordinary punishments, or restrained by repentance and reason, as the misplaced selfishness which endeavours to succeed by plan and calculation.

"As a consequence, these records, thus analysed, showed that the repetition of the crimes, and the return to prison by those whose crimes were of the physical nature, were more frequent than by those whose crimes were mostly connected with or aided by their intellect.

"These records alone showed, that of the inmates of the prisons reported, a thousand males were convicted and imprisoned somewhat less than two thousand times, while a thousand females were convicted and imprisoned nearly four thousand times.

"Of course the last inference is subject to the correction from other causes, such as that criminals, against property and persons are more migratory than the intemperate and lascivious, and therefore less likely to reappear at the same prison, and be recorded among the re-committals. Yet here is a very great difference of repetition, which must be charged to the difference of persistence of the origin of transgression.

"These are deductions from only a limited field of observation—from less than sixty-six thousand imprisonments.

"The number of facts may not be sufficient to establish the principle as to the comparative liability of the different sexes to different classes of crime, yet they are enough to indicate a further examination in the same field; and if the deductions here drawn be corroborated by a wider survey, then it will be for legislators and jurists to consider whether the Government and people should endeavour, by the same means, to repress and prevent crimes that have such widely-different origins, and to reform criminals whose temptations and power of resistance are so diverse."

In bringing this article to a conclusion, we would refer to one point more. In the course of the discussion upon a highly-valuable paper by Dr. Guy on *Statistical Methods and Signs*, exception was taken to his use of the term "statist" as the synonyme of and preferable term to "statistician," and the phrase "science of statistics." Dr. Guy "contended that there was a science of statistics, and that, practically, whenever we use the word 'statistics,' we mean the Science of States, irrespective altogether of the political discussions and differences prevailing between practical politicians." He further stated that his "reason for using the word 'statist' was that several of our old writers, Shakspeare, Beaumont and Fletcher, and others, have repeatedly used the words 'statist' and 'statism,' and evidently meant by them something analogous to statesmanship in politics; but there are two or three passages in the older writers which clearly show that the word 'statist' was used to characterize the scientific as distinguished from the practical politician or statesman. The word 'statistician,' according to our most common use of the term, would be such a person —a scientific speculator upon matters of government and statesmanship." He therefore thought he was justified in using the word "statist" in place of "statistician;" he also liked the word for its shortness, and for its being a good old English word. Dr. Farr agreed with Dr. Guy in the use of the word "statist," and he has used it in the sense of "statistician" throughout the present Report.

It is not without diffidence that we join issue with two such authorities, but we cannot avoid the conclusion that Dr. Guy, in the use of the word "statist" wrests it from its proper meaning. "Statist" was a term in use as signifying a statesman or politician in the ordinarily received sense of the term long before the term "statistics," or the need of that word, existed; and unless the meaning of the latter term be made co-extensive with the whole science and art of government, the former cannot be legitimately used to designate an adept in statistics. But Dr. Guy shows, in his own use of the word "statistics," and the phrase he would adopt as an equivalent—"Science of States"—that these terms are not synonymous with "politics." If we turn to the standard dictionaries of the English tongue, we find the respective meanings of "statist" and "statistician" accurately set forth. In the *Imperial Dictionary*, for example, we find—"*Statist* (from *state*), a statesman; a politician; one skilled in government."—"*Statistics*—that part of political science which is concerned in collecting and arranging facts illustrative of the condition and resources of a state or country, chiefly in relation to its extent, population, industry, health, and power, &c."—"*Statistician*—one versed in statistics." Unless the correctness of these definitions be disputed, we cannot well conceive that the word "statist" can be rightly substituted for "statistician," or "statistics" for "science of state." Lord Palmerston would be correctly described as a "statist," and Dr. Guy as a "statistician" (each also of the highest eminence), but would the converse hold good?

---

